# The exocyst in ciliogenesis

**DOI:** 10.1080/0886022X.2025.2519832

**Published:** 2025-06-23

**Authors:** Brennan Winkler, Kasey Lerner, Joshua H. Lipschutz

**Affiliations:** a Department of Medicine, Medical University of South Carolina, Charleston, SC, USA; b Department of Medicine, Ralph H. Johnson Veterans Affairs Medical Center, Charleston, SC, USA

**Keywords:** Polycystic kidney disease, renal failure, cilia, exocyst

## Abstract

The primary cilium is an organelle found on different cell types in many organs, and is important for human health including the kidney. Diseases due to abnormal or absent cilia are termed ciliopathies and ADPKD is one of the most common ciliopathies and the fourth leading cause of ESKD. The mechanisms of how primary cilia work remain incompletely understood. One particular axis of ciliary function that is especially unclear is the role of the highly-conserved eight-subunit exocyst trafficking complex, which is critically involved in transporting proteins from the trans-Golgi network to the cilium. The goal of this review article is to cover key aspects of exocyst function, how these are known to or are predicted to impinge on ciliary function, and to point out areas that need further research. The exocyst has been shown to be regulated by many different small GTPases of the Rho, Ral, Rab, and Arf families which likely give the exocyst specificity of function. The exocyst has been implicated in several intracellular signaling pathways involving the cilium including the MAPK and phosphoinositide pathways. The exocyst and its regulators have also been found in urinary extracellular vesicles suggesting that the exocyst may be involved in ‘urocrine’ signaling and repair following AKI. There is an urgent need to develop new strategies to address exocyst function in the context of cilia, which will greatly benefit our understanding of cilia as well as how disrupted exocyst function in cilia leads to disease, which, in turn, should lead to novel therapeutics.

## Introduction

The exocyst is a highly-conserved protein complex comprised of Exoc1-8 [1]. The exocyst was originally discovered in budding yeast by Novick, Field, and Schekman in a classic genetic screen and named because mutations inhibited exocytosis/secretion. The investigators reasoned that if the mother yeast cell could not secrete into the daughter cell, then the mother cells which were temperature-sensitive for secretion would become heavier during incubation at the non-permissive temperature of 37 °C. The heavier mutant cells could then be selected for using a Ludox density gradient. Using this system Exoc1-6 (also known as Sec3, Sec5, Sec6, Sec8, Sec10, and Sec15) were identified [2]. The final two additional exocyst subunits, Exoc7 and 8 (also known as Exo70 and Exo84) were identified in a subsequent screen [3]. Evidence points to the exocyst acting as a holocomplex. Mammalian homologues of all eight yeast exocyst proteins were identified in 1996 [4]. Exoc1-8 are hydrophilic proteins which interact to form a 19.5S complex associated with the plasma membrane [5]. Grindstaff et al. describe colocalization of the exocyst to the lateral membrane at cell-cell contacts with E-cadherin and ZO-1 in Madin-Darby canine kidney (MDCK) cells and specific distribution of these subunits to the apical-junctional complex on the lateral membrane domain as the cells polarize [5]. Additionally, the exocyst complex has been shown to interact with SNARE proteins at the plasma membrane for vesicle docking and fusion [6].

The exocyst complex has been shown to be necessary for ciliogenesis [7] and how this occurs will be the focus of this review article. Cilia are thin rod-like organelles extending outward from the basal body. During cell division, which is preceded by cilia disassembly, the basal bodies become centrioles. Cilia are primary (typically non-motile) or motile and contain a central axoneme composed of microtubules. Primary cilia respond to light, chemical, and mechanical cues [8–12]. Polycystins-1 and −2, the protein products of the PKD1 and PKD2 genes which are mutated in ADPKD, are important for mechanosensation by primary cilia found on kidney tubule cells, with polycystin-2 being a calcium channel [13–15]. Dozens of genes have been shown to be important for formation and/or function of primary cilia and result in PKD when mutated [16–18].

Understanding how the exocyst is regulated by small GTPases is crucial to appreciating how it is involved in ciliogenesis, which is the focus of the next section.

### Regulation of the exocyst by small GTPases

In yeast, mutants of individual exocyst proteins accumulate vesicles in their cells because the vesicles are not able to dock or fuse with the plasma membrane. The exocyst proteins localize to regions of active cell surface expansion, namely the bud tip at the beginning of the cell cycle and the mother-daughter cell connection during cytokinesis. This indicates that the exocyst is a central targeting and docking complex involved in directing vesicles from the trans-Golgi network to their precise sites of fusion including the primary cilium [19–22].

The exocyst plays a central role in exocytosis; however, how the exocyst itself is regulated is still being investigated. Given the complexity and ubiquity of the exocyst, one might imagine that it integrates many different inputs. Indeed, recent data from our Lab and others, demonstrates that multiple guanosine triphosphatases (GTPases), guanine nucleotide exchange factors (GEFs), and GTPase-activating proteins (GAPs) regulate the exocyst. GTPases, GEFs, and GAPs are families of proteins that allow for the bidirectional conversion between inactive guanosine diphosphates (GDPs) and active guanosine triphosphates (GTPs). Together, GTPases, GEFs, and GAPs integrate upstream regulatory inputs to influence various downstream effector outputs. As such, GTPases can be understood to be enzymatic on/off switches that are used to oversee cellular signaling cascades. Their evolutionary conservation across eukaryotes solidifies their fundamental role in regulating diverse biological processes in varying capacities.

The first GTPase recognized to interact with the exocyst was Rab8 (aka Sec4) [20]. A member of the Rab family, Rab8 is located on the surfaces of vesicular membranes and is required for normal post-Golgi secretory events in yeast. Rab8 associates directly in its GTP-bound form with the Exoc6 component (aka Sec15). The vesicle:Rab8-GTP:Exoc6 then binds to Exoc5 (aka Sec10), the central component that links the vesicle:Rab8-GTP:Exoc6 to the rest of the exocyst complex found at the targeting site [20]. These interactions culminate in the fusion and release of secretory vesicles contents into discrete plasma membrane domains. This initial observation of Rab8-exocyst dynamics prompted a deeper evaluation of the molecular networks responsible for triggering exocyst assembly and modulating its role in targeted exocytosis. Many other GTPases were subsequently identified, and those most relevant to ciliogenesis will be discussed in the following section.

### Regulation of the exocyst in ciliogenesis

Members of the Rab family, including Rab8 and Rab11, have been implicated in membrane trafficking and endosome recycling. Rab8 is activated by its GEF, Rabin8, which itself becomes activated *via* interaction with the GTP-bound form of Rab11. Immunofluorescence of these cellular components revealed that Rab8, Rabin8, and Rab11 concentrate in the projecting membrane, base, and basal body of cilia, respectively [23]. As such, a signaling cascade amongst Rab family members appears necessary for ciliary trafficking. Multiple studies have since shown the role of Rab8/Rabin8 in controlling membrane traffic to be integral to primary ciliogenesis [23,24]. Knodler et al. elucidated the role of Rab11 in ciliogenesis by demonstrating that inhibition of Rab11 by siRNA oligos led to shorter primary cilia in Infinity telomerase-immortalized human retinal pigment epithelial (hTERT-RPE1) cells when compared with wild-type controls [23]. Rab11 knockdown also led to a more proximal localization of Rab8 fluorescent staining in the shortened primary cilia of the hTERT-RPE1 cilia, though a complete loss of Rab8 was not observed [23]. In total, the coordination of Rab proteins appears to represent a strong link between the transport of cellular cargo from endosomal recycling (mediated primarily by Rab11) and vesicle fusion with the plasma membrane (mediated primarily by Rab8). Disruption of their communication impacts the completion of vesicular transport and cilia development.

The exocyst itself has been linked to endosome trafficking. Oztan et al. showed exocyst localization to multiple populations of endosomes and addition of function-blocking Exoc4 antibodies to streptolysin-O-permeabilized cells revealed exocyst requirements for several endocytic pathways including basolateral recycling, apical recycling, and basolateral-to-apical transcytosis. The latter was selectively dependent on interactions between the small GTPase Rab11a and Exoc6a and was inhibited by expression of the C-terminus of Exoc6a or down-regulation of Exoc6a expression using shRNA. These results suggested that the exocyst complex may be a multipurpose regulator of endocytic traffic directed toward both poles of polarized epithelial cells [25]. Despite this intriguing study virtually all subsequent studies have focused on the role of the exocyst in exocytosis.

Cdc42, a Rho family member, exhibited a direct association with Exoc1 (considered to be a spatial marker for the exocyst complex) and was revealed to be fundamental in linking this exocyst component with secretion of vesicles into the daughter cell in yeast [26]. In mammals, Cdc42 demonstrated unique activity alongside Exoc5 in tethering the exocyst to the primary cilium, where it appears to orchestrate the docking of vesicles carrying proteins for ciliogenesis in kidney tubule and photoreceptor cells [27,28]. A GEF of Cdc42, Tuba, is also necessary for ciliogenesis [29]. Initial investigation of Tuba knockdown in MDCK cells cultured in a collagen gel highlighted an impaired apical polarization process and failure of cilia formation. As an *in vivo* correlation to this experiment, individual morpholinos targeting Cdc42 and Tuba were injected into wild-type zebrafish embryos at the one-cell stage [29]. Tuba zebrafish morphants were found to have significant histological derangement of kidney development, such as highly disordered pronephric cilia and expanded glomeruli. The phenotypes of Tuba and Cdc42 morphants were similar and displayed a ‘ciliary mutant phenotype’ with tail curvature, hydrocephalus, small eyes, and cardiac edema. Co-injection of small amounts of Tuba and Cdc42 morpholinos, that alone had no effect, resulted in the above phenotypes indicating genetic synergy [29]. Therefore, Cdc42 and Tuba appear to communicate and operate within the same ciliogenic pathway.

Arl13B, a small GTPase of the ARF family, was shown to interact with the exocyst at the primary cilium [30]. More specifically, exocyst subunits Exoc2, Exoc4, and Exoc7 demonstrated a biochemical interaction with Arl13B in its GTP-bound (active) form. The possibility of a genetic interaction between Arl13B and the exocyst in ciliogenesis was investigated through the creation of *arl13b/exoc5* double-morphant zebrafish embryos. Functional studies of the zebrafish morphants showed a synergistic genetic interaction between Arl13B and Exoc5, with small amounts of the antisense morpholinos having no phenotypic effect but leading to tail curvature, small eyes, and pericardial edema when combined and injected at the one cell stage. Deletion of *arl13b* or *exoc5* in murine kidney cells produced a similar cilia-related phenotype, with decreased ciliogenesis in tubule epithelia and marked renal cyst formation in mice [30]. The fact that Arl13B is localized to the primary cilium suggests that Arl13B may be mediating a separate exocyst function within cilia. For example, Arl13B could be involved in the secretion of small extracellular vesicles (termed ectosomes) from the primary cilia, which is discussed in more detail in a subsequent section of this paper.

**Figure 1.**
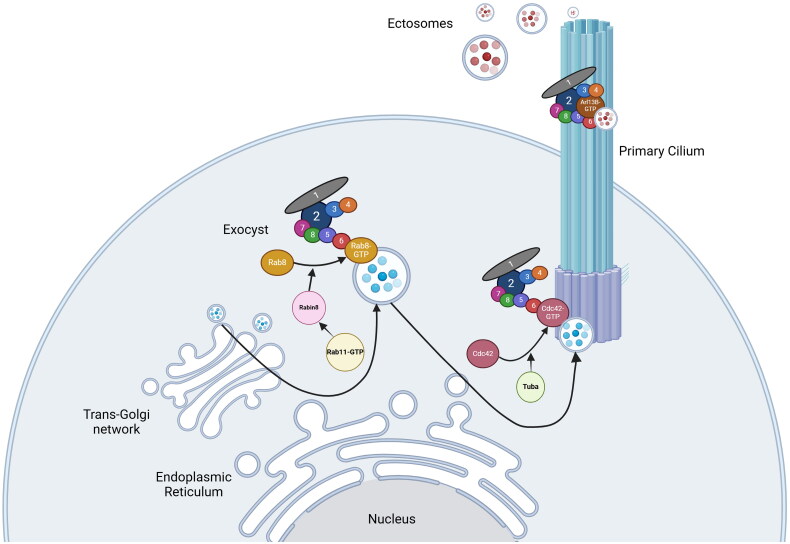
Model for exocyst-mediated trafficking of ciliary proteins. Genes are transcribed into mRNA in the nucleus and mRNA is translated into proteins in the endoplasmic reticulum. Proteins destined for the primary cilium are packaged in vesicles in the trans-Golgi network, and trafficked to the primary cilium by the exocyst complex. Small GTPases and GEFs, including Rab8, Rabin 8, Rab11, Cdc42, and Tuba, are involved in exocyst-mediated ciliary trafficking. Exoc5 is a central exocyst member as it links Exoc6 (bound to the vesicle *via* Rab8) and the rest of the exocyst complex. Once at the primary cilium the exocyst may be involved in ectocytosis of small extracellular vesicles mediated by small GTPases such as Arl13b. This figure was created using BioRender and we have received a license from BioRender for publication (*Winkler, B. (2025)* https://BioRender.com/r26n344).

Taken together, the exocyst’s encounters with members of the GTPase, GEF, and GAP superfamilies are among its most critical intracellular communications. The exocyst utilizes these networks to control vital cellular procedures, including vesicular trafficking, exocytosis, and establishment of polarity. As such, the functionality of the exocyst can be likened to that of a Swiss army knife, able to alter its activity in response to interactions with different molecular signals. The GTPases, GEFs, and GAPs involved in primary ciliogenesis are shown in Figure 1. Experimental manipulation of GTPase function and accessibility will almost certainly continue to aid in revealing the exocyst’s role as an effector of ciliogenesis and other cellular processes, as well as offer points of opportunity for targeted management of patients in clinical practice. Some of the available research tools are discussed next. The rest of the review will then focus on how these tools have been used to elucidate important molecular signaling pathways involved in the exocyst/cilia axis as well as in small extracellular vesicle (EV) production and content.

### Available research tools

Many labs have generated cell lines with knockdown, knockout, or overexpression of exocyst mutants. For example, we made stable Exoc5 overexpressing [31], knockdown [7], and ciliary targeting sequence mutant MDCK cells [32]. There are pharmacologic inhibitors of the exocyst available such as endosidin 2 which inhibits binding of exocyst components to the Exoc7 subunit [33]. There are also *in vivo* models such as zebrafish containing a CRISPR-generated mutation in Exoc5 [34] and mice with lox sites surrounding a crucial exon of Exoc5 [35] which, in conjunction with Cre-ERT2 mouse lines, allows for temporal and tissue specific knockout of Exoc5. These reagents have already been used to show the importance of the exocyst and cilia in organogenesis of the kidneys [30], heart [34], eyes [36], and ears [37].

### Mitogen activated protein kinase pathway

Perturbations in exocyst function lead to activation of multiple signaling pathways including the mitogen activated protein kinase (MAPK) pathway that is involved in primary ciliogenesis [38–40]. The MAPK pathway consists of a phosphorylation cascade among a series of proteins (Ras, Raf, MEK, ERK) that transduce extracellular mitogenic signals into tangible alterations in DNA transcription and cell activity. Upregulation of the MAPK pathway and its downstream effector, extracellular signal-regulated kinase (ERK), was observed following Exoc5 and Cdc42 knockdown/knockout, which provided initial evidence for its role in ciliogenesis [28,29,38]. Beyond its connection to Cdc42 and Exoc5, MAPK activity has been detected in other etiologies of dysfunctional primary cilia, such as polycystin-2 knockdown, nephronophthisis, and ADPKD, one of the most common ciliopathies [38,41,42].

A mechanism for the link between the MAPK pathway and the primary cilia was demonstrated by Sas et al. when they showed that bending of the primary cilium following flow increases intracellular calcium which activates PKC and, in turn, activates the Raf-1 kinase inhibitor protein (RKIP), a protein that normally keeps the MAPK pathway quiescent. When there is no primary cilium or the cilium is nonfunctional, intracellular calcium is low and RKIP is not generated allowing for activation of the MAPK pathway [43]. Similar to ADPKD cells, in Exoc5 KD cells, which lose primary cilia, we showed that intracellular calcium levels were low and did not respond to fluid flow, and MAPK levels were high [38].

With this in mind, manipulation of protein effectors within the MAPK/ERK pathway may offer a unique opportunity to slow the progression of or reverse disease processes related to cilia in human patients. Multiple studies have now shown that interference with this pathway *via* inhibition of phospho (active) ERK can reduce harmful propagation of cyst expansion and/or fibrosis [41,42]. Regarding ADPKD, upregulation of the MAPK pathway and its downstream effector, phosphorylated extracellular signal-regulated kinase (phospho-ERK) has been shown in Pkd2, one of two genes which when mutated lead to ADPKD [38]. Nephronophthisis, also a cystic kidney disease, is the most prevalent genetic cause of end-stage renal disease until age thirty when ADPKD then becomes the most common. Blockage of phospho ERK in murine nephronophthisis models (*pcy* and *inv)* through an oral inhibitor led to significant improvement in renal function and decreased cystogenesis [41,42]. Despite possible detrimental effects when overexpressed, the MAPK cascade remains essential to normal exocyst function and ciliogenesis. For example, Rabin8, mentioned above as a critical trafficking protein, can be activated by phospho ERK1/2 in response to EGF signaling [44]. Additionally, ERK1/2-mediated phosphorylation of Exoc7 has been found to promote exocyst assembly [45]. The phosphorylation of Rabin8 and Exoc7 by ERK1/2 suggests that a carefully regulated ERK response to extracellular signals may assist in the optimization of secretory activity and membrane trafficking involving the exocyst. Indeed, we showed that Exoc5 overexpression protects renal tubule cells from injury by activation of the MAPK pathway [46].

### Phosphoinositide pathway

Phosphatidylinositol phosphates (PIPs) play a large role in cell dynamics and are distributed throughout cell membranes, within organelles, and the cell periphery [47]. PIPs function in polarization, secretion, and cytoskeletal remodeling [48–50]. They have also been implicated in helping direct signaling cascades through spatial recruitment and are associated with exocyst function and primary cilia formation and maintenance [51–53]. The ciliary membrane is continuous with the plasma membrane but differs in its phospholipid composition. For example, phosphatidylinositol 4,5-bisphosphate (PI(4,5)P2) is significantly reduced toward the ciliary tip [54–56]. A possible explanation for this is the presence of specific PI(4,5)P2 degrading enzymes such as INPP5E localized in portions of the primary cilia [57]. Indeed, loss of function mutations in these enzymes cause ciliopathies in humans [58,59], indicating that the level of PI(4,5)P2 and its location in the ciliary membrane is functionally important. To determine the significance of this difference, Stilling et al. used chemically induced protein dimerization to rapidly synthesize or degrade PI(4,5)P2 selectively in the ciliary membrane. They observed ciliary shortening (disassembly) when PI(4,5)P2 was synthesized and increased ciliary length when PI(4,5)P2 was degraded [53]. Ciliary disassembly, which is necessary before cell cycle reentry can occur, required local actin polymerization, the Rho kinase Rac, aurora kinase A (AurkA) and histone deacetylase 6 (HDAC6) [60]. F-actin polymerization occurs following accumulation of PI(4,5)P2 in the primary cilia due to displacement of INPP5E driven by growth stimulation [60]. Activating ciliary receptors in the presence of dominant negative dynamin also increased ciliary PI(4,5)P2, increased the associated vesicle budding that requires ciliary PI(4,5)P2, and decreased ciliary length [53]. As noted, we previously showed that dynamin binding protein (Tuba) deficiency inhibits ciliogenesis and nephrogenesis *in vitro* and *in vivo* [29]. Stilling et al. hypothesized that changes in ciliary PI(4,5)P2 are a unifying point for ciliary membrane stability and turnover and that different stimuli increase ciliary PI(4,5)P2 to secrete vesicles and reduce ciliary length by a common pathway. The paucity of PI(4,5)P2 in the distal cilium therefore could ensure ciliary stability [53].

Ciliary localization of PIPs, especially PI(4,5)P2, affect the structural dynamics of primary cilia. In fact, PI(4,5)P2 appears to act as a substrate for components of the exocyst complex. In *S. cerevisiae*, only two exocyst subunits, Exoc1 (aka Sec3) and Exoc7 (aka Exo70), localize to the bud tip independently of actin cable–mediated transport and these subunits appear to localize *via* direct binding to Cdc42 and PI(4,5)P2 [26,61–63]. Pleskott et al. utilized coarse-grained molecular dynamics simulations to elucidate structural details of the interaction of yeast Exoc1 and Exoc7 with lipid bilayers containing PI(4,5)P2 [51]. They found that PI(4,5)P2 is coordinated by the positively charged pocket of the N-terminal part of Exoc1, which folds into a unique pleckstrin homology domain. Exoc7, on the other hand, interacts with the lipid bilayer by several binding sites distributed along Exoc7 [51]. They further found that interaction of Exoc7 with the membrane causes clustering of PI(4,5)P2 in the adjacent lipid leaflet and that PI(4,5)P2 is required for the correct positioning of the GTPase Rho1, a direct Exoc1 interactor, prior to the formation of the functional Rho1-exocyst-membrane assembly [51]. These structural data are supported by the findings of Liu et al. who demonstrated that the C-terminus of Exoc7 is necessary for its interaction with PI(4,5)P2 and recruitment of other components of the exocyst [50]. They demonstrated that Exoc7 associates with many different lipids found on the cell surface with different affinities, but shows the highest affinity for PI(4,5)P2 and PI(3,4,5)P3. They also showed that the interaction between Exoc7 and PI(4,5)P2 is critical for the docking and fusion of secretory vesicles from the Golgi [50].

Until recently, only partial crystal structures of five exocyst components had been obtained. These included near full-length yeast Exoc7, the C-terminal part of Exoc8 and Exoc3, and the N-terminal part of Exoc1 [64–68]. In addition, the crystal structures of near full-length mouse Exoc7, the C-terminal part of Drosophila Exoc6, zebrafish Exoc5, and the Ral-binding domain of rat Exoc8 were solved [69,70]. Interaction studies in yeast and mammalian cells indicated that there were two subcomplexes (Exoc1-4 forming subcomplex 1, and Exoc5-8 forming subcomplex 2) [71,72]. In 2018 Mei et al. determined a moderate-resolution cryoEM structure of the intact yeast exocyst complex and showed that the formation of the two subcomplexes is mediated by four helix bundles consisting of the conserved motifs from each of the subunits, with the two subcomplexes joining to form the octameric holocomplex [73]. Further protein structural work should allow researchers to define the extent to which exocyst complex members interact with different PIPs.

The relevant function of PIPs in conjunction with phosphatases and kinases will now be discussed in the context of GTPases with a focus on interactions with the exocyst involving ciliogenesis. Recently Maib et al. verified independent binding to PI(4,5)P2 by Exoc1 and Exoc7 [74]. In their studies they found that the kinase PIP5K1C, which converts PI(4)P to PI(4,5)P2, is recruited by the GTPase Arf6 where it acts on the PIPs following Arf6 activation. PI(4,5)P2 in conjunction with Arf6 recruits the exocyst to the membrane [74]. As discussed, we previously showed that another Arf family member Arl13b, which is mutated in the Joubert Syndrome ciliopathy, and the exocyst interact synergistically in ciliogenesis [30]. Another study looked at the interaction of Exoc1 and Exoc7 with GTPases Cdc42, Rho1, and Rho3 in the context of stearic acid-depleted phosphoinositides [48]. Lysophosphatidylinositol acyl transferase was knocked out in *S. cerevisiae* to achieve depletion of stearic acid. It was found that depletion of stearic acid-containing phosphoinositides negatively affected secretion. Yeast with phosphoinositides depleted of stearic acid had disrupted GTP/GDP ratios of Rho1, Rho3, and possibly Cdc42, thereby preventing interactions with Exoc1 and Exoc7 and activation of the exocyst complex [48]. We previously showed that Exoc5 and Cdc42 in zebrafish cooperate in ciliogenesis and that mice lacking Cdc42 in kidney tubule cells develop polycystic kidney disease [28]. In a follow-up study we examined the photoreceptor, which is a modified primary cilium, and showed in zebrafish that Cdc42 and Exoc5 act in the same pathway in photoreceptor and retinal development [27]. Based on the findings of Laquel et al. [48], the state of the acyl chains, involved in the recruitment of stearic acid, in PIPs could modulate the activity of small GTPases and subsequent interactions with the exocyst which, in turn, would affect ciliogenesis.

The location and metabolism of PIPs contributes to cell homeostasis. Garcia-Gonzalo et al. found that the ciliary membrane contains a particular phosphoinositide, PI(4)P, whereas a different phosphoinositide, PI(4,5)P2, is restricted to the membrane of the ciliary base [55]. The distribution is maintained by inositol polyphosphate-5-phosphatase E (Inpp5e), a ciliary phosphatase, and is important for Hedgehog (Hh) signaling through the primary cilia. Without Inpp5e ciliary PI(4,5)P2 levels are elevated and Hedgehog signaling is disrupted [55]. In Hh signaling, binding of sonic hedgehog (Shh) to the Patched (Ptc) receptor relieves Ptc inhibition on Smoothened (Smo) leading to ciliary accumulation of Smo. Gli transcription factors are then activated and translocate to the nucleus to produce Hh transcripts. Jiang et al. reported that Hh elevates production of PI(4)P [75]. Increased levels of PI(4)P promote, and decreased levels of PI(4)P inhibit, Hh signaling activity. They further found that PI(4)P directly binds Smo *via* an arginine motif, which allows for Smo phosphorylation and activation. Finally, Hh treatment increased the interaction between Smo and PI(4)P but decreased the interaction between Ptc and PI(4)P, suggesting that, in addition to promoting PI(4)P production, Hh regulates the pool of PI(4)P associated with Ptc and Smo [75]. Because Hh signaling has a profound effect on PI(4)P in the cilia, it is reasonable to hypothesize that Hh signaling plays a role in ciliary dynamics and exocyst function.

### The role of the exocyst and primary cilia in extracellular vesicle (EV) production and content

We hypothesize that the exocyst is involved in more than just delivering proteins to the primary cilia. If the exocyst was only involved in delivering proteins to the primary cilia we would expect to see it exclusively localize to the base of the primary cilia; however, we have noted in multiple organisms and cell types that the exocyst is found all along the length of the primary cilium [7,36,38]. The ‘urocrine signaling’ hypothesis posits that small secreted EVs contain proteins that transmit signals to distant cells. EVs can be produced through multivesicular bodies (termed exosomes) or *via* budding from primary cilia (termed ectosomes); however, the role of the exocyst and renal primary cilia in EV production and content remains understudied. We previously demonstrated that the entire exocyst complex and most of its known regulators are present in human urinary EVs [76]. We later showed that compared with control MDCK cells, EXOC5 overexpression increased EV production by ∼60% and Exoc5 KD decreased EV numbers by ∼30%. Proteomic analyses of isolated EVs from EXOC5 control, KD, and EXOC5-overexpressing MDCK cells revealed significant alterations in protein composition. To determine if this was an exocyst-specific effect, we also examined intraflagellar transport protein 88 (Ift88) KO and rescue cells. Ift88 KO cells lose primary cilia and have ∼60% fewer EVs than do Ift88 rescue cells. The protein content of EVs from the Ift88 KO and rescue cells was also very different, though the protein content of the Ift88 KO cells resembled that of the Exoc5 KD cells, therefore, we conclude that ectosome production content is dependent on the exocyst and the presence of primary cilia [77].

### Future perspectives

A major open question in the PKD field involves the identity of the ‘cilia-dependent cyst growth factor’. Virtually all forms of PKD are associated with defects in renal primary cilia structure, e.g., kidney-specific KO of Exoc5 [35,78] and KO of Ift88 [79], or function. For example, PKD1 and PKD2 KO cells that have normal appearing, but nonfunctional, cilia [17]. In 2013, it was shown that concomitant genetic removal of cilia in ADPKD mouse models suppressed cyst growth (Kif3a:Pkd1 and Ift20;Pkd2 double knockout mice) [80]. This result was surprising given that kidney cysts occur following complete removal of cilia by inactivation of Ift-related proteins [79] or following inactivation of polycystins in otherwise intact cilia. This phenomenon was confirmed by other groups using Ift88:Pkd1 and Ift88:Pkd2 double knockout mice which had shortened cilia, decreased kidney and liver cystogenesis, and reduced cell proliferation [81].

We recently showed very high levels of tryptophan following loss of cilia in Exoc5 KD, Exoc5 ciliary targeting sequence mutant (CTS-mut), and Ift88 KO cells as well as in proximal tubule-specific Exoc5 and Ift88 KO mice compared to their controls [82]. Metabolism of tryptophan to its downstream metabolite kynurenine in kidney cells occurs *via* indoleamine 2, 3-dioxygenases 1 and 2 (IDO1 and IDO2) [83]. Height adjusted total kidney volume (htTKV) is the best biomarker for ADPKD [84] and increased levels of kynurenine are associated with increased htTKV at baseline and percent change in htTKV over three years in children and young adults with ADPKD [85]. Grams et al. used data from the Modification of Diet in Renal Disease (MDRD) Study in which 28% of the patients had ADPKD and showed that kynurenate levels were highly associated with ADPKD compared to glomerular disease and chronic kidney disease (CKD) of other causes [86].

In Exoc5 KD, Exoc5 ciliary targeting sequence mutant (CTS-mut), and Ift88 KO cells tryptophan was increased, kynurenine was decreased, and IDO was decreased compared to control cells. This led to metabolically disadvantaged cells with defective mitochondria, increased reactive oxygen species (ROS), and decreased ATP synthase [82]. We hypothesize based on our data that tryptophan metabolism is the link between cilia and metabolism leading to cilia-dependent cyst growth, with cilia loss resulting in metabolic derangements that prevent the cell proliferation necessary to form cysts. If this is correct, then inhibitors of IDO may be novel treatments for ADPKD. This is supported by recent work showing elevated kynurenine and IDO1 levels in ADPKD Pkd1RC/RC mouse kidneys versus wildtype, increased IDO1 levels in ADPKD cell lines, reduced PKD severity with genetic Ido1 loss in Pkd1RC/RC mice, and less severe cystic kidney disease in Pkd1RC/RC mice and kidney-specific Pkd2-knockout mice following pharmacological inhibition of IDO1 [87].

## Summary

The exocyst is an important complex that targets and docks vesicles coming from the trans-Golgi network to various sites in cells, including the primary cilium. This complex appears to have many functions that are likely a result of different small regulatory GTPases acting on the exocyst. It is likely that the interplay between the exocyst, PIPs, and GTPases helps direct protein localization and ciliogenesis in different organisms. By manipulating the exocyst and/or its regulators and downstream targets, we may be able to treat a variety of ciliopathies.
